# A machine learning approach to predict mortality due to immune-mediated thrombotic thrombocytopenic purpura

**DOI:** 10.1016/j.rpth.2024.102388

**Published:** 2024-03-19

**Authors:** Mouhamed Yazan Abou-Ismail, Chong Zhang, Angela P. Presson, Shruti Chaturvedi, Ana G. Antun, Andrew M. Farland, Ryan Woods, Ara Metjian, Yara A. Park, Gustaaf de Ridder, Briana Gibson, Raj S. Kasthuri, Darla K. Liles, Frank Akwaa, Todd Clover, Lisa Baumann Kreuziger, Meera Sridharan, Ronald S. Go, Keith R. McCrae, Harsh Vardhan Upreti, Radhika Gangaraju, Nicole K. Kocher, X. Long Zheng, Jay S. Raval, Camila Masias, Spero R. Cataland, Andrew D. Johnson, Elizabeth Davis, Michael D. Evans, Marshall Mazepa, Ming Y. Lim

**Affiliations:** 1 Division of Hematology and Hematologic Malignancies, Department of Internal Medicine, University of Utah, Salt Lake City, Utah, USA; 2 Division of Epidemiology, Department of Internal Medicine, University of Utah, Salt Lake City, Utah, USA; 3 The Department of Medicine, Johns Hopkins University, Baltimore, Maryland, USA; 4 Department of Medicine, Emory University, Atlanta, Georgia, USA; 5 Department of Medicine, Wake Forest University, Winston-Salem, North Carolina, USA; 6 Department of Medicine, University of Colorado, Denver, Colorado, USA; 7 Department of Pathology and Laboratory Medicine, University of North Carolina, Chapel Hill, North Carolina, USA; 8 Geisinger Medical Laboratories, Danville, Pennsylvania, USA; 9 Department of Pathology and Laboratory Medicine, Emory University, Atlanta, Georgia, USA; 10 Department of Medicine, University of North Carolina, Chapel Hill, North Carolina, USA; 11 Department of Medicine, East Carolina University, Greenville, North Carolina, USA; 12 Department of Medicine, University of Rochester, Rochester, New York, USA; 13 St Charles Healthcare, Bend, Oregon, USA; 14 Versiti, Milwaukee, Wisconsin, USA; 15 Department of Medicine, Medical College of Wisconsin, Milwaukee, Wisconsin, USA; 16 Department of Medicine, Mayo Clinic, Rochester, Minnesota, USA; 17 Department of Medicine, Cleveland Clinic, Cleveland, Ohio, USA; 18 Department of Internal Medicine, University of Texas Southwestern Medical Center, Dallas, Texas, USA; 19 Department of Medicine, University of Alabama at Birmingham, Birmingham, Alabama, USA; 20 Department of Pathology and Laboratory Medicine, University of Kansas Medical Center, Kansas City, Kansas, USA; 21 Institute of Reproductive Medicine and Developmental Sciences, University of Kansas Medical Center, Kansas City, Kansas, USA; 22 Department of Pathology, University of New Mexico, Albuquerque, New Mexico, USA; 23 Baptist Health South Florida, Miami, Florida, USA; 24 Department of Medicine, The Ohio State University, Columbus, Ohio, USA; 25 Department of Laboratory Medicine and Pathology, University of Minnesota, Minneapolis, Minnesota, USA; 26 Department of Medicine, University of Minnesota, Minneapolis, Minnesota, USA; 27 Clinical & Translational Science Institute, University of Minnesota, Minneapolis, Minnesota, USA

**Keywords:** artificial intelligence, machine learning, mortality, statistical models, thrombotic thrombocytopenic purpura

## Abstract

**Background:**

Mortality due to immune-mediated thrombotic thrombocytopenic purpura (iTTP) remains significant. Predicting mortality risk may potentially help individualize treatment. The French Thrombotic Microangiopathy (TMA) Reference Score has not been externally validated in the United States. Recent advances in machine learning technology can help analyze large numbers of variables with complex interactions for the development of prediction models.

**Objectives:**

To validate the French TMA Reference Score in the United States Thrombotic Microangiopathy (USTMA) iTTP database and subsequently develop a novel mortality prediction tool, the USTMA TTP Mortality Index.

**Methods:**

We analyzed variables available at the time of initial presentation, including demographics, symptoms, and laboratory findings. We developed our model using gradient boosting machine, a machine learning ensemble method based on classification trees, implemented in the R package gbm.

**Results:**

In our cohort (*n* = 419), the French score predicted mortality with an area under the receiver operating characteristic curve of 0.63 (95% CI: 0.50-0.77), sensitivity of 0.35, and specificity of 0.84. Our gradient boosting machine model selected 8 variables to predict acute mortality with a cross-validated area under the receiver operating characteristic curve of 0.77 (95% CI: 0.71-0.82). The 2 cutoffs corresponded to sensitivities of 0.64 and 0.50 and specificities of 0.76 and 0.87, respectively.

**Conclusion:**

The USTMA Mortality Index was acceptable for predicting mortality due to acute iTTP in the USTMA registry, but not sensitive enough to rule out death. Identifying patients at high risk of iTTP-related mortality may help individualize care and ultimately improve iTTP survival outcomes. Further studies are needed to provide external validation. Our model is one of many recent examples where machine learning models may show promise in clinical prediction tools in healthcare.

## Essentials

• Predicting immune thrombotic thrombocytopenic purpura (iTTP) mortality may help individualize care.
• A previous prediction model had suboptimal performance using the United States Thrombotic Microangiopathies database.
• Our machine-learning model predicted acute iTTP mortality with a cross-validated area under the receiver operating characteristic curve of 0.77.
• If externally validated, the United States Thrombotic Microangiopathies iTTP Mortality Index (ustma.org/calculator) may aid clinicians.

## Graphical abstract

**Figure:**
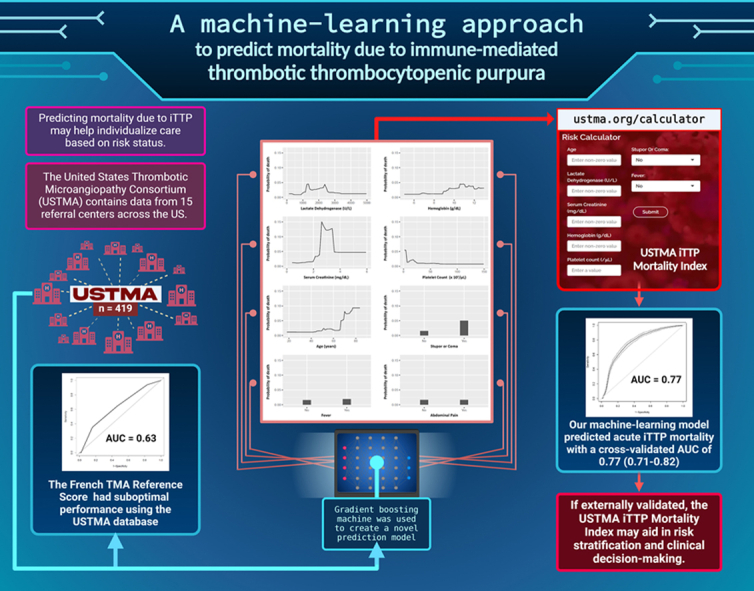

## 1 Introduction

Immune-mediated thrombotic thrombocytopenic purpura (iTTP) is a rare, life-threatening thrombotic microangiopathy (TMA) caused by a severe acquired deficiency of ADAMTS13 [1]. iTTP was associated with a mortality rate greater than 90% in early reports [2,3] as a result of microvascular thrombosis that can lead to tissue ischemia and multiorgan failure [4]. Treatment with therapeutic plasma exchange (TPE) and immunosuppression significantly lowers iTTP-related mortality rates [3,5]. Yet, despite improvements in diagnosis and treatment of iTTP, the most recent mortality rate estimates remain around 5% to 10% in the past decade [[6], [7], [8], [9], [10]].

Predicting the risk of iTTP-related mortality may potentially help individualize treatment based on disease severity. In 2012, the French Thrombotic Microangiopathy (TMA) Reference Score was developed as a predictive model for acute TTP mortality and incorporates age, lactate dehydrogenase (LDH) level, and cerebral involvement [8]. The score was validated in the French TMA registry; however, external validation in a large United States population has not been performed and thus the score’s applicability is unclear in this population. The United States Thrombotic Microangiopathy (USTMA) registry was established in 2014 and contains demographics and outcome data on 771 patients diagnosed with iTTP from 15 high-volume TTP referral centers across the nation between 1985 and 2019.

Therefore, we utilized the USTMA registry to validate the French TMA Reference Score and to subsequently develop a novel mortality prediction model. Initially, we used the Least Absolute Shrinkage and Selection Operator to create a prediction model, which demonstrated poor performance after performing cross validation [11]. We then utilized machine learning to create a novel prediction model. Recent advances in machine learning have generated growing interest and promise in its applicability to medicine, particularly for the development of outcome prediction models [[12], [13], [14]]. Traditional predictive models are typically developed from regression models with a small number of candidate variables. In contrast, machine learning methods can explore a large number of predictor variables, flexibly modeling their relationships to the outcome as potentially nonlinear and utilizing interactions. These models can then be made publicly available for clinical use as online calculators [13,15]. These advanced capabilities make machine learning methods suitable for analyzing large numbers of variables from a registry such as ours to predict clinical outcomes.

## 2 Methods

### 2.1 Patient selection

We included all participants who had available data on their first episode of iTTP (*n* = 419) from the USTMA registry (*n* = 771) between 1985 and 2019. Patients who did not have any available information on their presenting episode were excluded. The study cohort included participants with iTTP diagnosis based on the presence of thrombocytopenia (platelet count < 100 × 10^3^/μL), microangiopathic hemolytic anemia (defined as hemoglobin [Hgb] levels of less than the lower limit of normal with schistocytes on the peripheral blood smear), and either ADAMTS13 activity <10% or ADAMTS13 activity <20% with an anti-ADAMTS13 inhibitor or antibody. For participants diagnosed before the ADAMTS13 assay was available (2006), the iTTP diagnosis was based on the clinical course and absence of alternative causes; we could not exclude these participants due to the low number of fatal outcomes in our database. We were not able to calculate the PLASMIC score [16], a tool that can distinguish between patients with and without severe ADAMTS13 deficiency, as some of the necessary variables were not part of the USTMA Registry. We instead compared the baseline variables of those with and without ADAMT13 confirmation. We defined acute iTTP mortality as death caused by iTTP occurring from the time of presentation until 30 days of last TPE. The outcome mortality was not adjudicated or investigated, and was determined by each participating center and entered into the USTMA database. Data availability was dependent on whether the local investigator entered the data into the USTMA registry. All local IRBs approved the USTMA registry studies.

### 2.2 Statistical analysis

All study variables were summarized and stratified by mortality status, and were analyzed as either continuous variables (eg, laboratory values and age) or categorical variables (eg, presence of symptoms and sex). Continuous variables were summarized as mean, standard deviation (SD), median, interquartile range (IQR), and range. Categorical variables were summarized as count and percentage. Missingness information was provided for each variable stratified by mortality status. We similarly compared ADAMTS13-confirmed vs ADAMTS13 not tested on the top 5 influential variables from our gradient boosting machine (GBM) model.

### 2.3 Validation of French TMA Reference Score

We assigned risk scores of 0 to 4 to patients in our registry according to the French TMA Reference Score based on age, LDH level, and the presence of neurologic symptoms. We assessed the performance of the risk score in our dataset using the area under the receiver operating characteristic curve (AUC) and its 95% CI.

### 2.4 Development of the USTMA TTP Mortality Index

#### 2.4.1 Machine learning method: gradient boosted trees

We used GBM, a machine learning ensemble method based on classification trees, implemented in the R package gbm [17], to develop our prediction model for acute mortality. GBM has the flexibility of incorporating complex interactions among variables as well as enabling nonlinear relationships between predictor variables and outcomes. Furthermore, it can make predictions in the presence of missing data. Thus, we consider all baseline variables for prediction without imputation. We included 18 baseline variables available at the time of initial presentation, including age, sex, symptoms, and laboratory measures (Table 1). The tuning parameters did not have a big impact on results, so we used a fixed set of parameters for all models (see Supplementary Appendix for details).

**Table 1:** Patient and disease characteristics corresponding to first episode by mortality.

Variable	Dead (*n* = 24)	Alive (*n* = 395)
Demographic variables		
Age (y)^a^		
Mean (SD)	50.3 (17.6)	43.3 (15.3)
Median (IQR)	51.2 (40.3, 59.5)	41.6 (32.3, 54.8)
Range	(12.0, 78.9)	(3.6, 83.7)
Age < 18	1 (4.1%)	15 (4.2%)
Age ≥ 60	6 (25%)	57 (15.6%)
Sex^a^		
Female	13 (54.2%)	283 (71.6%)
Male	11 (45.8%)	112 (28.4%)
Race		
White/Caucasian	10 (41.7%)	145 (37.3%)
Black/African American	13 (54.2%)	230 (59.1%)
Other	1 (4.2%)	14 (3.6%)
Symptom variables^a^		
Fever	7 (29.2%)	74 (18.7%)
Chest pain	0 (0%)	39 (9.9%)
Abdominal pain	7 (29.2%)	97 (24.6%)
Fatigue	8 (33.3%)	128 (32.4%)
Petechiae or easy bruising	3 (12.5%)	104 (26.3%)
Dark urine	3 (12.5%)	53 (13.4%)
Presence of any neurologic symptoms	15 (62.5%)	227 (57.5%)
Confusion	7 (29.2%)	105 (26.6%)
Seizure	1 (4.2%)	25 (6.3%)
Memory deficit	1 (4.2%)	19 (4.8%)
Stupor or coma	4 (16.7%)	21 (5.3%)
Headache	4 (16.7%)	91 (23%)
Stroke or TIA	6 (25%)	86 (21.8%)
Presence of any symptoms	23 (95.8%)	353 (89.4%)
Laboratory variables		
ADAMTS13 activity—mean (SD)	3.1 (6.1)	2.3 (6.9)
Median (IQR)	0.0 (0.0, 0.0)	0.0 (0.0, 0.0)
Range	(0.0, 17.0)	(0.0, 53.0)
Inhibitor present	11 (45.8%)	255 (64.6%)
Antibody present	5 (20.8%)	34 (8.6%)
Hemoglobin (g/dL)^a^—mean (SD)	9.8 (2.2)	8.4 (1.9)
Median (IQR)	9.5 (7.8, 11.2)	8.1 (7.1, 9.4)
Range	(6.8, 14.3)	(3.5, 13.9)
Platelet count^a^ (× 10^3^/μL)—mean (SD)	18.3 (14.3)	21.6 (24.1)
Median (IQR)	16.0 (11.0, 19.5)	14.0 (9.5, 26.0)
Range	(2.0, 66.0)	(2.0, 233.0)
Lactate dehydrogenase (U/L)^a^—mean (SD)	1954.6 (1475.6)	1646.1 (1395.2)
Median (IQR)	1455.0 (1262.0, 2370.0)	1223.0 (779.0, 1911.0)
Range	(397.0, 7101.0)	(138.0, 9349.0)
Creatinine (mg/dL)^a^—mean (SD)	2.0 (1.0)	1.5 (1.2)
Median (IQR)	1.8 (1.2, 2.5)	1.2 (0.9, 1.7)
Range	(0.4, 4.8)	(0.4, 16.0)
Peak troponin—mean (SD)	1.0 (1.4)	1.8 (4.0)
Median (IQR)	0.5 (0.2, 1.0)	0.4 (0.1, 1.5)
Range	(0.1, 3.8)	(0.0, 26.1)

Missing values (number among dead/number among alive): age = 0/29, aged ≥60 years = 0/29, aged <18 years = 0/29, race = 0/6, ADAMTS13 activity = 7/62, hemoglobin level = 5/18, platelet count = 1/8, lactate dehydrogenase = 7/22, creatinine = 2/26, and peak troponin = 18/269.

a Considered for our prediction model.

Our reported GBM model was developed on the full dataset, but the performance of the model was assessed using cross-validation as described below. We reported relative influences of all variables selected by the GBM model, where relative influence is defined as the percentage of prediction error reduced by each variable relative to the total error reduced by all selected variables. We made our reported GBM model available in the form of an online calculator (www.ustma.org/calculator).

#### 2.4.2 Assessment of model performance

Repeated (100 times) 10-fold cross-validation was used to evaluate model performance by estimating AUC, as well as sensitivity, specificity, and positive predictive value (PPV) at selected thresholds.

On each iteration, the data were divided into 10 folds, where each fold was used as the test set to evaluate the GBM model developed on the remaining 9 folds of data (training). The results from the 10 test folds were combined to calculate the AUC (ie, on the full data set, *n* = 419), and then, the AUCs were averaged across the 100 iterations. The 95% CI for the AUC was determined from the 2.5th and 97.5th percentiles of its distribution. We fixed the tuning parameters across all folds and iterations as they had little impact on our model results (see Supplementary Appendix for details). Two methods were used to identify thresholds in the training dataset: the Youden Index (sensitivity + specificity - 1) and the smallest predicted death probability of those who actually died. These approaches were then applied to the test data to determine sensitivity, specificity, and PPV. We also evaluated our model using a calibration plot to compare observed versus predicted risk of death, and we examined the impact of class imbalance (ie, a 5.7% death rate) on model development and performance (see Supplementary Appendix for details). To assess the performance of our model specifically on patients with ADAMTS13-confirmed iTTP diagnosis, we calculated the AUC by excluding those without ADAMTS13 confirmation from each test set.

### 2.5 Data sharing statement

Agreement to share publication-related data and data sharing statement: data utilized for this study come from the USTMA TTP registry. Data and R code, including (deidentified) individual participant data, will be made available to others with written request to the USTMA Consortium upon publication of the manuscript. To request access, formal request must be made in writing to the corresponding PI and include the proposed analysis. Approval of the analysis will be granted after review by participating members of the USTMA Consortium and full consensus on the terms of use (including review of the analysis and final manuscript as well as authorship arrangements) and signed data access agreement with the University of Minnesota.

## 3 Results

### 3.1 Patient characteristics

There were 24 deaths (5.7%) that occurred during the patients’ first iTTP episode in our cohort of 419 patients. The median time to death was 8 days (IQR: 3, 17). Baseline patient and disease characteristics based on survivor status are summarized in Table 1.

Since some of the variables needed to calculate the PLASMIC score were not part of the dataset, we could not compare the PLASMIC scores of patients with ADAMTS13 confirmation (*n* = 362) with those without it (*n* = 57). We instead compared their baseline characteristics (Supplementary Table S1), and did not find significant differences except for higher age and hemoglobin in the ADAMTS13-confirmed group. All patients were treated with TPE and 81% received steroids. Only 28% received rituximab, 10% received other immunosuppression, and none received caplacizumab as our study predates its approval.

### 3.2 Validation of the French TMA Reference Score

**Figure 1:**
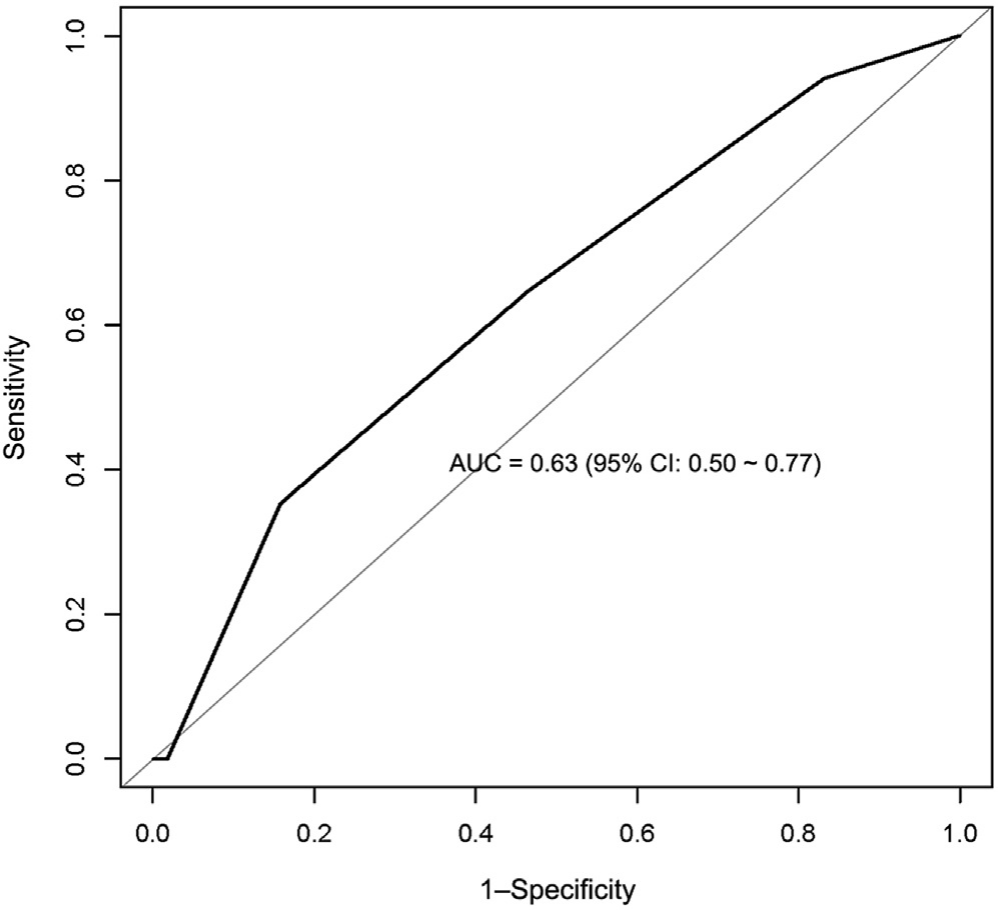
Receiver operating characteristic curve showing the performance of the French TMA Reference Score in predicting iTTP mortality in our dataset. iTTP, immune-mediated thrombotic thrombocytopenic purpura; TMA, thrombotic microangiopathy.

The French TMA Reference Score had an AUC of 0.63 (95% CI: 0.50-0.77; Figure 1) predicting mortality from acute iTTP in the USTMA cohort. Using the recommended cutoff of ≥3, the sensitivity was 0.35, specificity was 0.84, and PPV was 0.1 in our population.

### 3.3 Development of the USTMA TTP Mortality Index

**Figure 2:**
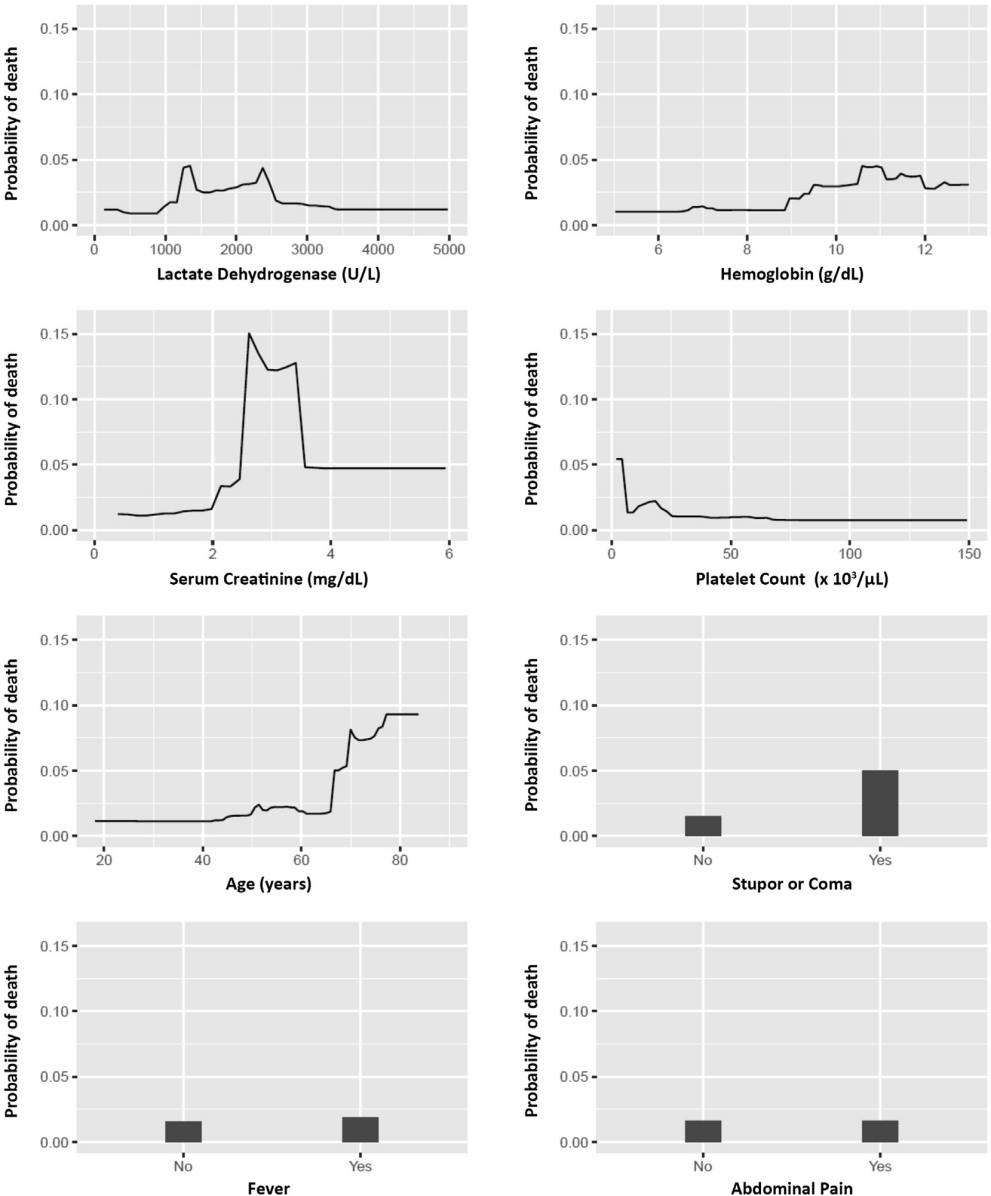
Partial dependence plots of the variables used in our model.

All variables from Table 1 were considered as candidate predictors for constructing a mortality prediction model except for race, peak troponin (due to high missingness), and ADAMTS13 antibody/inhibitor (due to reporting inconsistencies). Among the 18 variables considered for our prediction model, age and LDH had the highest missingness at 7% each. Our GBM model selected 8 variables to predict acute mortality: LDH, hemoglobin, creatinine, age, platelet count, stupor/coma, fever, and abdominal pain at the time of initial presentation. Their relative influence is shown in Table 2. Due to its minimal relative influence, abdominal pain was not included in the final model. The partial dependence plots are shown in Figure 2. While the probability of death is low (generally <0.05) across most values of the selected variables, there is a complex relationship between serum creatinine and death, where values between 2.5 mg/mL and 3.5 mg/mL were associated with a notable probability increase. We also see an increase in the probability of death for patients aged 65 years and older. While the GBM model did not remove observations with missing values, we note that there was 17% missingness among the variables selected by our GBM model (ie, *n* = 348 complete case observations).

**Table 2:** The 8 predictors selected by GBM, shown in order of highest to lowest relative influence.

Variable	Relative influence (%)
Lactate dehydrogenase (U/L)	39.76
Serum creatinine (mg/dL)	18.69
Age (years)	18
Hemoglobin (g/dL)	14.36
Platelet count (×10^3^/μL)	4.45
Stupor or coma	3.56
Fever	0.97
Abdominal pain^a^	0.22

a Due to its minimal influence, abdominal pain was not included in the final model.

### 3.4 Performance of the machine learning model

**Figure 3:**
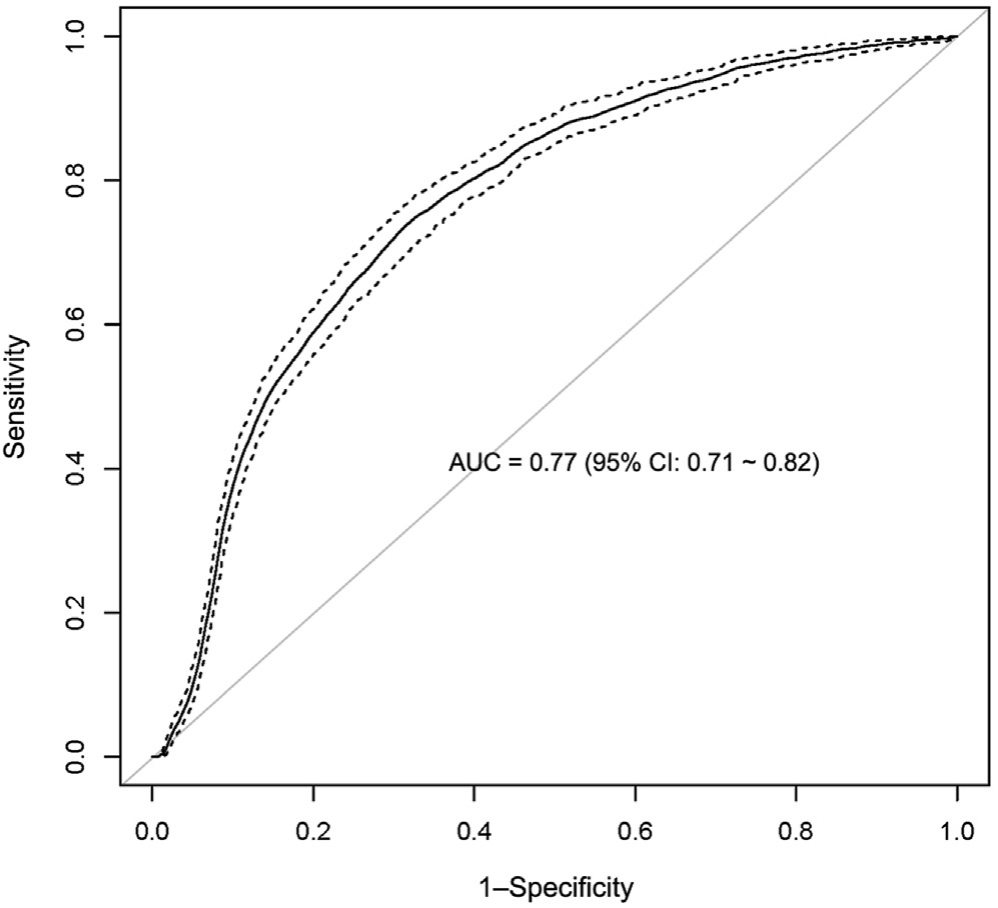
Receiver operating characteristic curve of the USTMA iTTP Mortality Index showing the performance of our final model in predicting iTTP mortality. iTTP, immune-mediated thrombotic thrombocytopenic purpura.

The machine learning model had an AUC of 0.77 (95% CI: 0.71-0.82) **(**Figure 3). When applied to the full dataset, the threshold using the smallest predicted death probability of those who actually died was 0.0427, which we used as the cutoff for “high-risk” disease. The threshold of the predicted mortality that maximized the Youden Index was 0.0908, which we used as the cutoff for “very high-risk” disease. The sensitivities, specificities, and PPVs of both cutoffs using the full data and cross-validated methods are reported in Table 3. Using these cutoffs, we risk-stratified the patients based on predicted mortality risk as follows:

**Table 3:** Sensitivity, specificity, and positive predictive value of the 2 cutoff points in the USTMA TTP Mortality Index.

Threshold method	Threshold	Full data			Repeated 10-fold cross-validation		
		Sensitivity	Specificity	Positive predictive value	Sensitivity	Specificity	Positive predictive value
Minimum observed risk among deceased	0.0427	1.00	0.709	0.173	0.642	0.757	0.138
Maximum Youden Index	0.0908	0.917	0.896	0.349	0.500	0.867	0.185

Standard risk: predicted risk < 4.27%.

High risk: predicted risk ≥ 4.27% and < 9.08%.

Very high risk: predicted risk ≥ 9.08%.

A calibration plot is provided in the Supplementary Appendix that shows our model performs best for the risk range of 5% to 10%. It tends to underestimate risk for patients with a risk of death of >20%, but this does not impact our calculator’s interpretations of standard risk, high risk, and very high risk. The observed death per risk group is shown in Table 4.

**Table 4:** Observed death and predicted risk on full data.

Predicted risk	Total number	Number of deaths (%)
Standard risk (<0.0427)	279	0
High risk (0.0427-0.0908)	77	2 (2.6%)
Very high risk (>0.0908)	63	22 (34.9%)

For patients with a confirmed ADAMTS13 diagnosis, our model had an AUC of 0.76 (95% CI: 0.69-0.83).

To increase accessibility of our model, we created an online calculator available at www.ustma.org/calculator.

## 4 Discussion

Our study is the largest iTTP registry study to evaluate predictors of iTTP-related death to date and the first to use GBM methodology to develop a mortality prediction model. Throughout the past 3 decades, attempts to identify predictive variables using traditional analyses in the literature have repeatedly demonstrated inconsistent results [8,[18], [19], [20], [21], [22]], likely due to the rarity and heterogeneity of iTTP biology. In our previous logistic regression analysis [11], most variables trended toward, but did not independently reach, statistical significance in predicting risk of mortality, again suggesting the unreliable nature of individual variables in predicting iTTP mortality.

In our cohort, the French TMA Reference Score demonstrated poor ability to predict iTTP mortality with an AUC of 0.63 (95% CI: 0.50-0.77) as evidenced by the AUC’s 95% CI lower bound of 0.50. In comparison, the AUC in the French TMA study was 0.77. This may be related to population differences as well as differences in health care systems in France and the United States. Additionally, the time gap between our study and the study period of the French score (2000-2011) may have played a role due to improvements in the management of iTTP since then, or perhaps even a possible impact of the score itself in the recognition of iTTP since its publication. It is not unusual for newer prediction models to perform worse on external datasets utilizing different patient populations, especially for a disease as rare and heterogenous as iTTP.

With a heterogenous, rare disease such as iTTP, the use of machine learning is a promising approach. Our study demonstrated that the machine learning model of the USTMA Mortality Index had improved performance, with an AUC of 0.77 (95% CI: 0.71-0.82). Our model can be used as an online calculator (www.ustma.org/calculator), which is not yet ready for clinical practice as it lacks external validation. The calculator classifies patients with a predictive risk of <4.27% as “standard risk” for acute mortality, 4.27% to 9.08% as “high risk”, and >9.08% as “very high risk”. Both cutoffs demonstrated acceptable specificity (74% and 86%). However, the sensitivity was low (63% and 50%). This means that our model could not “rule out” the possibility of death. This is likely both a consequence of the low number of fatal outcomes in our cohort and other limitations of our dataset described below, as well as the heterogenous and unpredictable nature of iTTP.

The USTMA Mortality Index’s acceptable specificity for predicting mortality suggests that it may be useful in identifying patients who are at higher risk of mortality. However, due to its low sensitivity, the model may not be suitable for identifying “low-risk” patients, and every patient should be considered to be at risk of death. There are several ways by which identifying high-risk patients may impact clinical care in the future, if it can be validated in external dataset(s). Tailoring iTTP management based on individual risk may improve overall outcomes in high-risk patients. For example, such patients may be considered for more intensive therapy beyond the current standard of care. Certain approaches have been described as potentially beneficial in life-threatening iTTP, such as the use of twice-daily TPE [[23], [24], [25]], earlier introduction of rituximab, or more aggressive immunosuppression with pulse cyclophosphamide [23]. Additionally, high-risk patients would benefit from greater supportive care at an intensive care unit, closer clinical monitoring, and vigilant evaluation of cardiac and renal functions. Those with high-risk disease in the community setting may also be considered for urgent transfer to a center with both expertise in management of iTTP and access to novel therapeutics such as caplacizumab, which may offer a mortality benefit [26,27].

The variables selected by our prediction model have been previously reported in some studies, but not others: these include renal dysfunction [[18], [19], [20], [21], [22]], older age [8,18,19], cerebral involvement [8,19,20], high LDH [8], and low platelet count [28]. Troponin has been previously described as a predictor of worse prognosis [7], but we chose not to consider it due to high missingness (more than half of our cohort). While ADAMTS13 inhibitor/antibody levels have also been suggested as poor prognostic factors [[29], [30], [31]], we were unable to reliably use them in our analysis due to inconsistency of their reporting in our retrospective registry. Regardless, their utility in real-life mortality risk prediction in the acute setting is limited because the test results are rarely available on presentation. Our model also showed that higher Hgb concentration was independently associated with worse iTTP survival, which has not been reported before. Of note, this finding reflects presenting Hgb and does not reflect the true degree of hemolysis since baseline Hgb levels prior to iTTP onset in our patients are unknown. One hypothesis is that nondecreased Hgb levels on admission may have led to an underrecognition of iTTP and an ensuing delay in diagnosis and empiric treatment, which increases the risk of mortality beyond the first 24 hours [32]. We were unable to test this hypothesis because the time from symptom onset until empiric treatment is not documented in our registry. Another potential explanation for this association is that higher baseline Hgb, and in turn, higher erythrocyte counts may in fact directly exacerbate microvascular thrombosis in iTTP due to interactions between erythrocytes and ultralarge von Willebrand factor (ULVWF). Previous evidence suggests that erythrocytes may be essential to the pathogenesis of microangiopathies by actively binding to ULVWF [33,34], and it can be postulated that higher erythrocyte levels may further exacerbate microvascular thrombosis. Further studies on the contribution of erythrocytes to TMA-related organ ischemia and mortality are needed.

A major limitation of our study is the low number of fatal outcomes (5.7% of patients), which is consistent with recent randomized trials [35], but lower than historically reported. This may be related to improvements in early recognition and urgent treatment of iTTP based on education efforts, clinical diagnostic scores [16], and perhaps better access to care, which aided the early recognition of iTTP and its empiric management in recent years. The fact that all patients in our cohort were treated at expert referral centers may have also lowered mortality rates and may have also created a selection bias. It is also possible that fatal iTTP cases in more remote decades were missed. The low number of fatal outcomes limited our ability to develop a robust prediction model and potentially led to an overoptimistic assessment of model performance. While machine learning methods are appreciated for their flexible modeling capabilities, this flexibility means that they are sensitive to noisy data and prone to overfitting, particularly in datasets with low event rates. This may have also impacted the performance of the logistic regression model, which may have performed better in a different setting. The partial dependence plots in our model indicate a complex relationship between serum creatinine and death, which could be evidence of overfitting. As a result, our model may not perform as well in practice as it performed on our dataset. While the GBM machine learning approach is able to handle missing data, there was notable missingness in our selected model (17%). Another limitation of our study was including patients diagnosed prior to the availability of the ADAMTS13 assay, which may have led to the inclusion of patients that had TMAs other than iTTP. We could not exclude those patients due to the low number of fatal outcomes in our database. However, the majority of our patients did have ADAMTS13 confirmation, and we feel confident that all cases were likely correctly diagnosed for several reasons. We did not find major differences in the baseline characteristics of those with and without ADAMTS13 confirmation. Most notably, serum creatinine level was similar in that group, and the median of 1.0 g/dL strongly suggests against Shiga toxin or complement-mediated hemolytic uremic syndrome as the cause for TMA (Supplementary Table S1). Furthermore, when repeating the 10-fold analysis on those with ADAMTS13 confirmation only, the model had a near identical AUC performance. Another major limitation of our study is that our patient cohort spans 3 decades, during which mortality outcomes changed significantly with time and patients from more recent years likely had better outcomes due to improved disease recognition and treatment [36]. During our study period, all patients received TPE, which has been the standard of care for iTTP treatment. However, we did not have information on any potential delays in TPE initiation in earlier decades, which could have impacted mortality. It is also possible that with the emergence of novel iTTP therapies, the future standard of care may evolve to differ significantly from that of our patient population (eg, caplacizumab use with or without TPE). For that reason, the accuracy of our model may likely be reduced in the contemporary setting as practice patterns and disease recognition continue to improve with time [36], and our model would have to be reassessed. A final and notable limitation is that we did not have any external validation data for our prediction model. Future studies are needed to validate our prediction model before it can reliably be used in a clinical setting, particularly given the potential for overfitting.

In conclusion, the USTMA Mortality Index machine learning model had acceptable performance in predicting mortality due to acute iTTP in the USTMA registry and had good specificity in identifying high-risk patients. However, it did not perform well at identifying low-risk patients. Identifying patients at high-risk of iTTP-related mortality may help individualize care, intensify treatment for these patients, and ultimately improve iTTP survival outcomes. Our model is one of many recent examples where machine learning techniques are promising in creating new avenues in future healthcare and advancing clinical prediction tools. However, since machine learning methods have a tendency to overfit and yield overly optimistic performance assessments in datasets with low event rates, further studies are needed to externally validate our prediction tool before it is used to guide clinical decision making. Overall, death from iTTP in the modern era remains infrequent, yet very difficult to predict, even with the use of machine learning.
