# Supplementary material for: A machine learning approach to predict mortality due to immune-mediated thrombotic thrombocytopenic purpura
Source: Res Pract Thromb Haemost. 2024 Mar 19;8(3):102388. doi: 10.1016/j.rpth.2024.102388 (PMC11033197; doi:10.1016/j.rpth.2024.102388)
Supplement: Supplementary material [file mmc1.docx]

**Supplementary Material**

**Table of Contents**

| **Supplementary Appendix** | | 2 |
| --- | --- | --- |
| **Supplementary Table S1.** Baseline demographics and laboratory variables stratified by availability of ADAMTS13 confirmation status. | 5 | |
| **Supplementary Table S2.** Fatal outcomes at initial presentation in patients with and without ADAMTS13 confirmation | | 6 |

**Supplementary Appendix**

This Appendix includes additional details regarding model building and evaluation.

**Tuning parameters**

We performed a grid search across the following tuning parameters:

shrinkage/learning rate (0.0005, 0.001, 0.005, 0.01), bag fraction = c (0.5, 0.6, 0.7), interaction depth (1, 2, 3), number of trees (2000 ~10000 depending on the learning rate), and minimum observations per node (10, 20), assessing performance using the 10-fold cross validation built in to the gradient boosted machine (GBM) function. While the best set of tuning parameters differed each time we repeated this process, the cross validated errors were similar, and thus the tuning parameters had little impact on model construction. This was likely due to the modest size of our data set (N=419) with only 24 deaths relative to the 18 candidate predictors. Since the tuning parameters did not have a big impact on results, we used a fixed set of parameters for all models: a relatively slow learning rate (shrinkage = 0.005), a large number of trees (n.trees = 2000), a bag fraction of 0.7 an interaction depth of 2, and a minimum of 10 observations per node.

**Accounting for class imbalance**

Due to the rarity of our outcome (5.7%), we repeated the model building process with observations re-weighted with weights inversely proportional to the prevalence of their observed outcome (ie, subjects that died were weighted roughly 16.5 times of the survivors). We also assessed performance using repeated (100 times) 10-fold cross validation. We then compared the area under the receiver operating characteristic curve (AUC) and area under the precision-recall curve (AUPRC) of this model to our original model.

Results:

The original model had a cross validated AUC of 0.77 and AUPRC of 0.133. AUPRC is expected to be lower than AUC because the lowest value for AUC is 0.5, whereas the lowest value for the AUPRC is the fraction of positives, which is 0.057.

The weighted model (red line in figure below) has a lower AUC of 0.73 and a higher AUPRC of 0.145. Although this model was more precise at a lower sensitivity, it could not maintain this precision when trying to identify the majority of the deaths. Since sensitivity is of higher priority in our case, we opted to use the original model.

**Single Split Results**

Our reported model described in the main text is based on applying GBM to our full data set, and our assessment of this model’s performance is based on repeated (100 times) 10-fold cross-validation because we were concerned that a single split approach would lead to biased results in our small data set. However, we also examined the resulting model and its performance under a single split approach. Specifically, we split the data into 80% (training) and 20% (testing), and used the training data to develop the model, then test the performance on the testing data. When we supply all 18 candidate predictors, the model developed on the training data had 11 selected predictors versus 8 selected by the original model. This model had an AUC of 0.76 on the 20% test data. If we only supplied our 8 selected variables as candidate predictors, the model developed on the training data kept all 8 variables. This model also had an AUC of 0.76 on the test data. These results are similar to the cross validated AUC of 0.77 for our reported model.

**Model calibration**

We assessed the calibration of our model using the calibration plot below, where individuals were grouped into bins of predicted risks of 0~0.025, 0.025~0.05, 0.05~0.1, 0.1~0.2, and >0.2. For each group the observed proportion of death with binomial confidence intervals were plotted against the average predicted risk of death.

The plot shows that our model prediction is most accurate for the middle risk range (0.05~0.1) and underestimates risk for individuals with very high risk (> 0.2), which is encouraging since it is most challenging to discriminate people in the middle range. Since our prediction thresholds are >0.0427 for high risk and >0.0908 for very high risk, high risk is still easily identified by our model even with an underestimated risk.

**Supplementary Table S1.** Baseline demographics and laboratory variables stratified by availability of ADAMTS13 confirmation status.

| Variable | ADAMT13 confirmed (N=362) | ADAMTS13 not tested (N=57) | P-value |
| --- | --- | --- | --- |
| Age (years) | | | |
| Mean (SD) | 44.6 (15.5) | 38.2 (14.3) | 0.003^t^ |
| Median (IQR) | 43.0 (33.0, 55.2) | 37.8 (27.2, 46.9) | - |
| Sex | | | |
| Female | 256 (70.7%) | 40 (70.2%) | 0.93^c^ |
| Hemoglobin (g/dL) | | | |
| Hemoglobin - Mean (SD) | 8.5 (2.0) | 7.6 (1.7) | - |
| Median (IQR) | 8.2 (7.2, 9.6) | 7.2 (6.4, 8.9) | 0.010^e^ |
| Platelet count (x 10^3^/µL) | | | |
| Mean (SD) | 20.8 (23.6) | 26.2 (24.4) | - |
| Median (IQR) | 14.0 (9.0, 24.0) | 16.5 (11.0, 31.8) | 0.06^e^ |
| Serum Creatinine (mg/dL) | | | |
| Mean (SD) | 1.5 (1.2) | 1.4 (0.9) | - |
| Median (IQR) | 1.2 (0.9, 1.8) | 1.0 (0.8, 1.7) | 0.23^e^ |
| Lactate dehydrogenase (U/L) | | | |
| Mean (SD) | 1664.3 (1420.1) | 1611.0 (1169.4) | - |
| Median (IQR) | 1256.0 (795.5, 1904.5) | 1239.0 (788.5, 2340.5) | 0.97^e^ |

Missing values: Age=28/1, Hemoglobin=1/22, Platelets=0/9, Creatinine=4/24, LDH=7/22.

^t^ T-test, ^c^ Chi-squared test, ^e^ Exact wilcoxon rank sum test.

**Supplementary Table S2.** Fatal outcomes at initial presentation in patients with and without ADAMTS13 confirmation

|  | **ADAMTS13 confirmed (n=362)** | **ADAMTS13 not confirmed (n=58)** |
| --- | --- | --- |
| **Fatal outcome** | 17 (4.7%) | 7 (12.1%) |
